# *Coxiella burnetii*, the Agent of Q Fever, Replicates within Trophoblasts and Induces a Unique Transcriptional Response

**DOI:** 10.1371/journal.pone.0015315

**Published:** 2010-12-14

**Authors:** Amira Ben Amara, Eric Ghigo, Yannick Le Priol, Catherine Lépolard, Suzana P. Salcedo, Emmanuel Lemichez, Florence Bretelle, Christian Capo, Jean-Louis Mege

**Affiliations:** 1 Unité de Recherche sur les Maladies Infectieuses Tropicales et Emergentes, CNRS-IRD UMR 6236, IFR 48, Université de la Méditerranée, Marseille, France; 2 IMTSSA, Transcriptomic Platform, Parc du Pharo, Marseille, France; 3 Centre d'Immunologie de Marseille-Luminy, Université de la Méditerranée, UMR 6546, Marseille, France; 4 INSERM Unité 895, Equipe 6, C3M, Microbial Toxins in Host Pathogen Interactions, Nice, France; Institut de Pharmacologie et de Biologie Structurale, France

## Abstract

Q fever is a zoonosis caused by *Coxiella burnetii*, an obligate intracellular bacterium typically found in myeloid cells. The infection is a source of severe obstetrical complications in humans and cattle and can undergo chronic evolution in a minority of pregnant women. Because *C. burnetii* is found in the placentas of aborted fetuses, we investigated the possibility that it could infect trophoblasts. Here, we show that *C. burnetii* infected and replicated in BeWo trophoblasts within phagolysosomes. Using pangenomic microarrays, we found that *C. burnetii* induced a specific transcriptomic program. This program was associated with the modulation of inflammatory responses that were shared with inflammatory agonists, such as TNF, and more specific responses involving genes related to pregnancy development, including EGR-1 and NDGR1. In addition, *C. burnetii* stimulated gene networks organized around the IL-6 and IL-13 pathways, which both modulate STAT3. Taken together, these results revealed that trophoblasts represent a protective niche for *C. burnetii*. The activation program induced by *C. burnetii* in trophoblasts may allow bacterial replication but seems unable to interfere with the development of normal pregnancy. Such pathophysiologocal processes should require the activation of immune placental cells associated with trophoblasts.

## Introduction

Q fever is a widespread zoonosis caused by *Coxiella burnetii*, an intracellular gram-negative bacterium recognized as a potential category-B bioweapon. Human primary infection is asymptomatic or present as isolated fever, hepatitis, or pneumonia. It spontaneously resolves but may become chronic endocarditis in patients with valve lesions, arterial aneurysm, or prosthesis or in those who are immunocompromised [1]. During pregnancy, Q fever may result in obstetric complications including spontaneous abortion, intrauterine growth retardation or fetal death, and premature delivery. In addition, pregnant women exhibit an increased risk of Q fever endocarditis even in the absence of underlying valvular lesions [2]. This phenomenon has been reproduced in C57BL/6 mice, where infection followed by repeated pregnancies was shown to result in spontaneous abortion, perinatal death and endocarditis in some animals [3]. In cattle, sheep and goats, *C. burnetii* infection is often unapparent [4], [5], but there is increasing evidence that it is associated with spontaneous abortion and stillbirth [5], [6]. The placentas of infected animals contain up to 10^9^ organisms/g of tissue, and it is likely that heavily infected placentas contaminate the environment at the time of parturition, leading to the persistence of viable organisms in the soil for several months [7]. In addition, aerosols from the secretions and excretions of ruminants are the major source of contamination for humans [8].

*C. burnetii* is known to survive in the myeloid cells of humans and experimentally infected mice by resisting their natural microbicidal activity [9], [10]. The cell types infected by *C. burnetii* in humans during pregnancy are unknown. Immunocytochemical studies have shown that in cows the *C. burnetii* antigen is present within trophoblasts, especially along the base of chorionic villi and in the intervillous spaces in the placentas of aborted fetuses [11]. Using in situ hybridization, it has been shown that *C. burnetii* is found both within trophoblasts and free in placenta debris from ruminant abortions [12]. Inoculation of *C. burnetii* into pregnant goats leads to an initial infection of trophoblasts of the choriallantoic membrane that precedes a massive bacterial colonization of the placenta, followed immediately by spontaneous abortion [13]. Most studies of pathogens exhibiting placental tropism, such as human cytomegalovirus [14], *Toxoplasma gondii* [15] or *Brucella abortus* [16], have used placental tissues but were unable to define the cell targets of the infection.

Although the precise mechanisms by which infection compromises pregnancy are largely undefined, it is clear that excessive inflammation at the maternal interface could play a critical role. Placental tissue contains trophoblasts and cells with inflammatory and immunomodulatory potential that can be mobilized in response to infection. Among the inflammatory mediators produced by cells at the materno-fetal interface, Tumor Necrosis Factor (TNF) may affect both placental function and the local immune response. Studies have shown that TNF is critical for both fetal development and placental function [17]. TNF is beneficial during the pre-implantation period; it prevents development of offspring with structural anomalies, controls the differentiation of extravillous trophoblasts, balances trophoblast turnover and renewal, and stimulates uterine activity. However, exaggerated production of TNF induces apoptosis of trophoblasts, inhibits the production of human chorionic gonadotropin and the subsequent trophoblast fusion, and controls invasive trophoblast differentiation. These deleterious events that occur during bacterial and viral infections [18] may lead to obstetrical complications such as preeclampsia, premature rupture of membranes, preterm labor and spontaneous abortion.

Because the cellular distribution of *C. burnetii* in the placenta is not known, we chose to investigate if this bacterial pathogen could infect trophoblasts, which are essential for successful pregnancy. We show that *C. burnetii* infects and replicates within BeWo trophoblasts, a human trophoblastic cell line. Further, we show that *C. burnetii* stimulation induces a transcriptional activation program consisting of both *C. burnetii*-specific features and an inflammatory response similar to TNF. Such findings demonstrate that trophoblasts are a target for *C. burnetii* and respond to the bacteria with an activation program that could account for pregnancy complications resulting from Q fever.

## Results

### *C. burnetii* replication in BeWo trophoblasts

**Figure 1:**
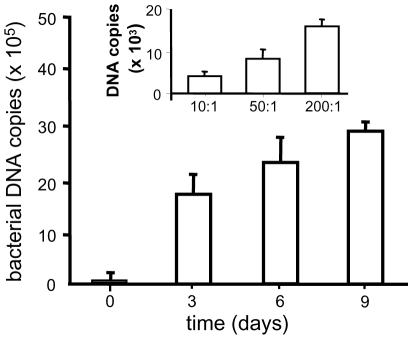
Intracellular fate of *C. burnetii.* BeWo trophoblasts were incubated with different concentrations of *C. burnetii* (10, 50 and 200 bacteria/cell) for 4 h and extensively washed to remove free organisms (insets). They were then cultured for 9 d. The number of bacterial DNA copies was determined using qPCR. The results represent the mean ± SEM of 3 experiments.

Because *C. burnetii* is found in the placenta of aborted fetuses [11], [19], we investigated the ability of *C. burnetii* organisms to infect BeWo trophoblasts. We extended the previously described procedure for establishing macrophage infection by *C. burnetii* [20] to BeWo trophoblasts. Cells were infected with different doses of bacteria per cell for 4 hours and washed to remove any unbound bacteria; this time was defined as day 0. We found that BeWo cells were infected by *C. burnetii* in a dose-dependent manner (Fig. 1, inset), as demonstrated by qPCR analysis performed on the *C. burnetii com1* gene [9]. Using immunofluorescence, we determined that roughly 50% of infected cells contained one or two bacteria (data not shown), a level of infection similar to that previously described in human monocytes [21]. In subsequent experiments, an infecting dose of 200 bacteria per cell was used. We found that BeWo trophoblasts supported substantial bacterial replication (Fig. 1). At day 0, the number of bacterial DNA copies was 14±0.5×10^2^; this number increased ninety fold at day 3 post-infection and steadily increased thereafter to reach 2.6±0.8×10^6^ at day 9 post-infection. These results clearly demonstrated that BeWo cells were pertinent to the study of *C. burnetii* replication in trophoblasts.

**Figure 2:**
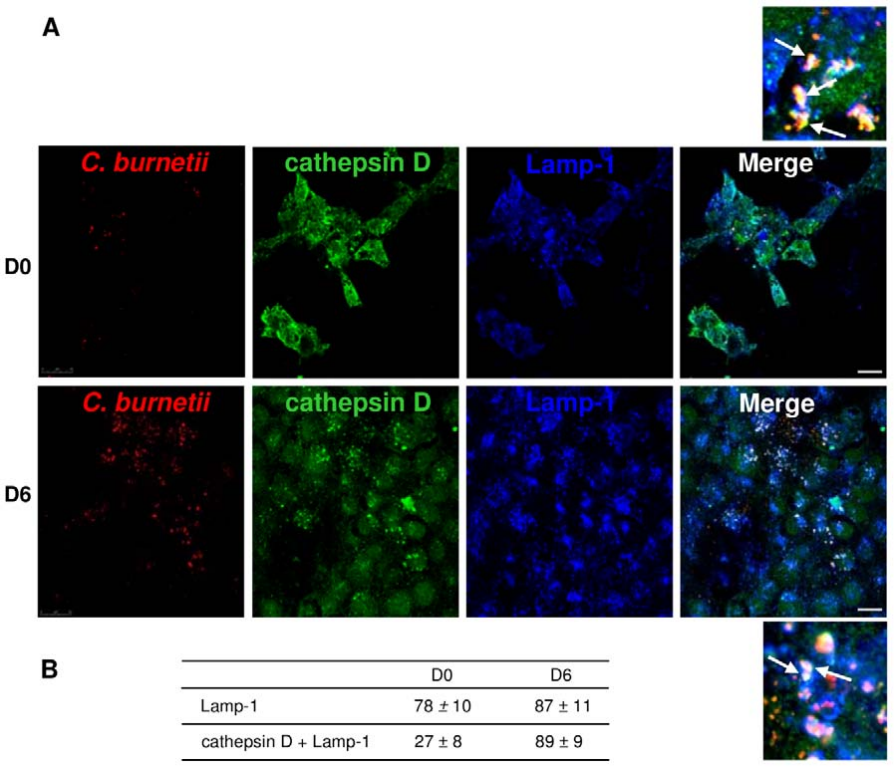
Intracellular localization of *C. burnetii.* Infected BeWo cells were labeled with anti-*C. burnetii* (Alexa 546), anti-cathepsin D (Alexa 488) and anti-Lamp-1 (Alexa 647) Abs and analyzed by laser scanning confocal microscopy. A, The co-localization of *C. burnetii* (red) with Lamp-1 (blue) and cathepsin D (green) is demonstrated by merging the respective fluorescent images. The co-localization of bacteria with both Lamp-1 and cathepsin D appears as white (see arrows), whereas the co-localization of bacteria with only Lamp-1 appears as yellow (see arrows). B, The percentage of bacteria that co-localized with Lamp-1 and cathepsin D was recorded. The results represent the mean ± SD of approximately 60 cells for each experimental condition. Scale bars represent 5 µm. D0 and D6 are day 0 and day 6 post-infection, respectively.

Because the survival of *C. burnetii* in macrophages is based on the defective maturation of *C. burnetii*-containing phagosomes into phagolysosomes [20], we investigated the characteristics of the *C. burnetii*-containing compartment in BeWo trophoblasts using confocal microscopy (Fig. 2). At day 0, the majority of organisms co-localized with lysosomal-associated membrane protein (Lamp)-1, a marker of late endosomes and lysosomes [20]; only 27±8% of organisms co-localized with cathepsin D, a marker of lysosomes [20]. At day 6 post-infection, more than 80% of organisms co-localized with both cathepsin D and Lamp-1. Taken together, these results showed that the replication of *C. burnetii* in BeWo trophoblasts was associated with the presence of the bacteria in phagolysosomes.

### *C. burnetii*-induced transcriptional program

**Figure 3:**
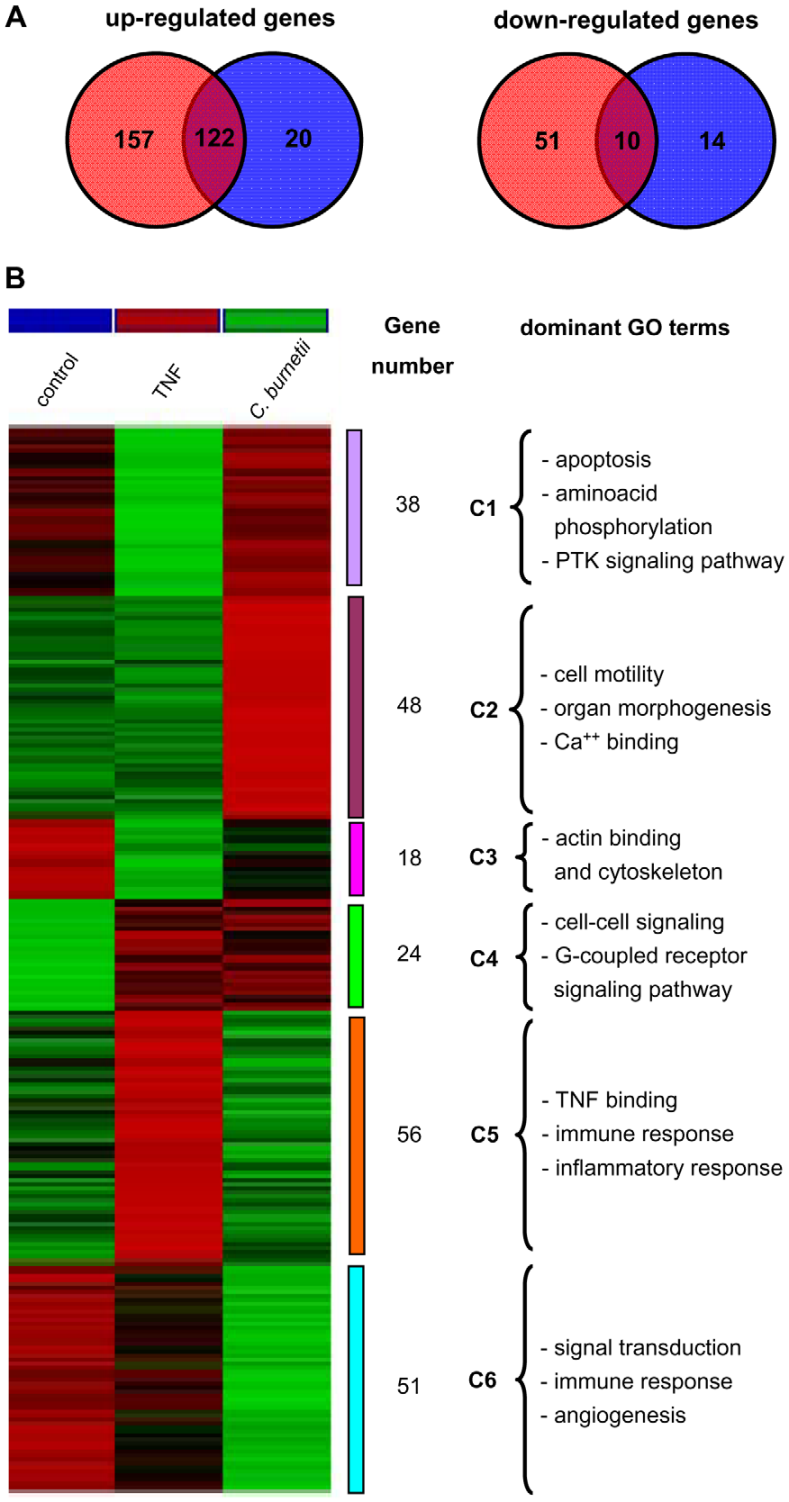
Venn diagram and hierarchical clustering analysis. BeWo cells were incubated with *C. burnetii* (200 bacteria/cell) or TNF (10 ng/mL) for 6 h. Total RNA was extracted and, after cyanin-3 incorporation, hybridized on chips representing 31,054 genes. Only genes that were expressed with a P value <0.05 and a confidence value >0.3 in at least one condition were included in the analysis. A, The number of genes modulated in response to *C. burnetii* (red) and TNF (blue) stimulation is indicated. Intersections showed that 122 and 10 genes were up-regulated and down-regulated, respectively, by both *C. burnetii* and TNF stimulation. B, Data were analyzed using hierarchical clustering and are represented by a color gradient (Z-score) ranging from blue (down-modulation) to yellow (up-regulation). Left: unstimulated cells (NS); middle: TNF-stimulated cells; Right: *C. burnetii*-stimulated cells. The GO analysis was performed using two filters: 1. GO term level 2< gene <11; 2. when GO term is represented by more than 3 genes on the chip and contains more than 2 genes of the study set. The number of genes in every biological process is presented.

**Figure 4:**
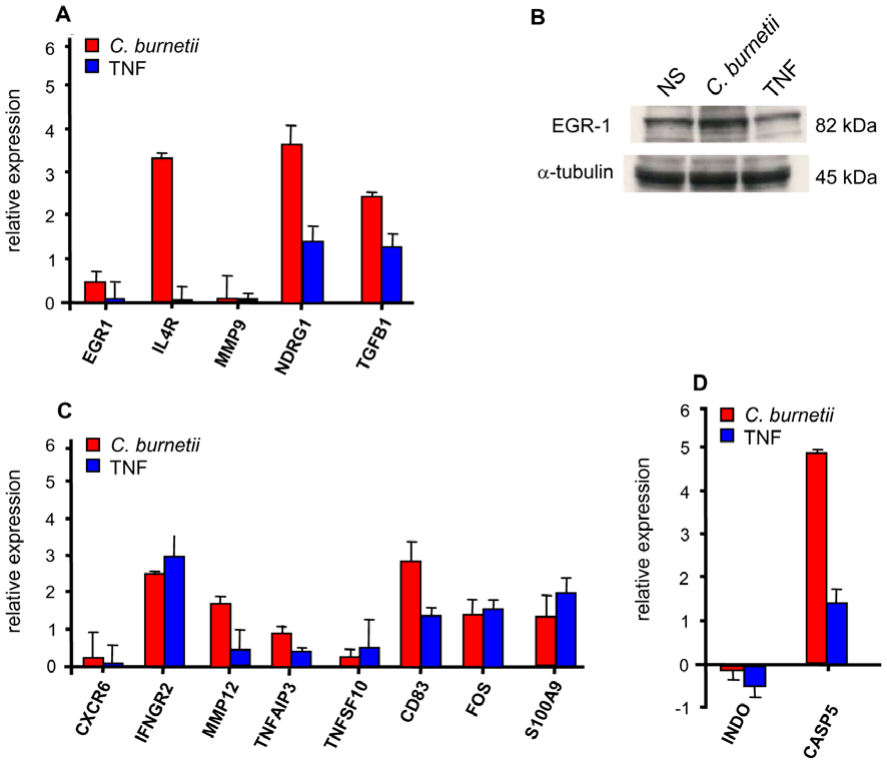
Real time RT-PCR and western blot analysis. BeWo cells were stimulated with *C. burnetii* for 6 h. A, C and D; RNA was extracted and real time RT-PCR was performed. Results expressed in relative expression (stimulated vs. unstimulated conditions) represent the mean ± SEM of 3 independent experiments. B, Western blot analysis was performed using specific mAbs for α-tubulin and EGR-1, and bands were revealed by chemoluminescence. Their intensity was determined by densitometry and represented the mean of three experiments.

A whole-genome microarray approach was used to define the transcriptional signature of *C. burnetii* infection in BeWo trophoblasts. We found that 340 genes were significantly modulated after a 6-hour stimulation with *C. burnetii* (Fig. 3A); they consisted of 279 up-regulated genes with fold changes (FCs) ranging from 1.4 to 4.96 (Table S1) and 61 down-regulated genes with FCs ranging from -1.4 to -1.83 (Table S2). The clustering analysis of the 340 genes modulated in response to *C. burnetii* stimulation revealed a specific profile that could be organized in 6 clusters using GO terms (Fig. 3B). Cluster 1 contained 38 genes up-regulated in response to *C. burnetii* stimulation and showed enrichment for genes involved in apoptosis and kinase signaling. Cluster 1 also showed that the expression of the monocarboxylate transporter SCL16A3 (a solute carrier family 16 protein), which is known to play a role in pre-implantation [22], increased in response to *C. burnetii* stimulation. In cluster 2, 48 genes corresponding to cell motility and calcium binding GO terms were enriched in response to *C. burnetii* stimulation. These included genes encoding EGR1 (early growth response 1), IL4R (interleukin 4 receptor), MMP9 (matrix metallopeptidase 9), NDRG1 (N-myc-downstream regulated 1) and TGF-β1 (Transforming Growth Factor). The increased expression of these genes was confirmed by real-time RT-PCR (Fig. 4A). In addition, the expression level of EGR-1 protein in *C. burnetii*-stimulated BeWo cells was determined by western blot. The results showed that the amounts of EGR-1 were higher in stimulated BeWo cells than in control cells (Fig. 4B); autoradiograms quantified by scanning densitometry revealed a relative enrichment of 1.47±0.23. In clusters 3 and 4, *C. burnetii* induced a moderate modulation of 18 and 24 genes, respectively (Fig. 3B). These genes showed enrichment for actin-binding and cell-cell signaling proteins. In cluster 5, 56 genes were modulated with enriched GO terms for immune and inflammatory responses. Among these genes, the expression of the genes encoding CXCR6, IFNGR2 (interferon-gamma receptor 2, also known as IFNγ transducer 1), MMP12 (also known as macrophage elastase), TNFAIP3 (tumor necrosis factor alpha-induced protein 3), TNFSF10 (TNF ligand superfamily member 10), CD83, FOS (FBJ murine osteosarcoma viral oncogene homolog) and S100A9 (S100 calcium-binding protein A9) were up-regulated. This increased expression was confirmed using real-time RT-PCR (Fig. 4C). Interestingly, the analysis of cluster 6 (representing 51 modulated genes) showed that *C. burnetii* depressed the expression of the INDO gene (indoleamine-pyrrole 2,3-dioxygenase) and increased expression of CASP5 (apoptosis-related cysteine peptidase). These changes were confirmed by real time RT-PCR (Fig. 4D). Taken together, these results showed a specific transcriptional program in BeWo trophoblasts infected with *C. burnetii*.

### Specific transcriptional networks stimulated by *C. burnetii*

**Figure 5:**
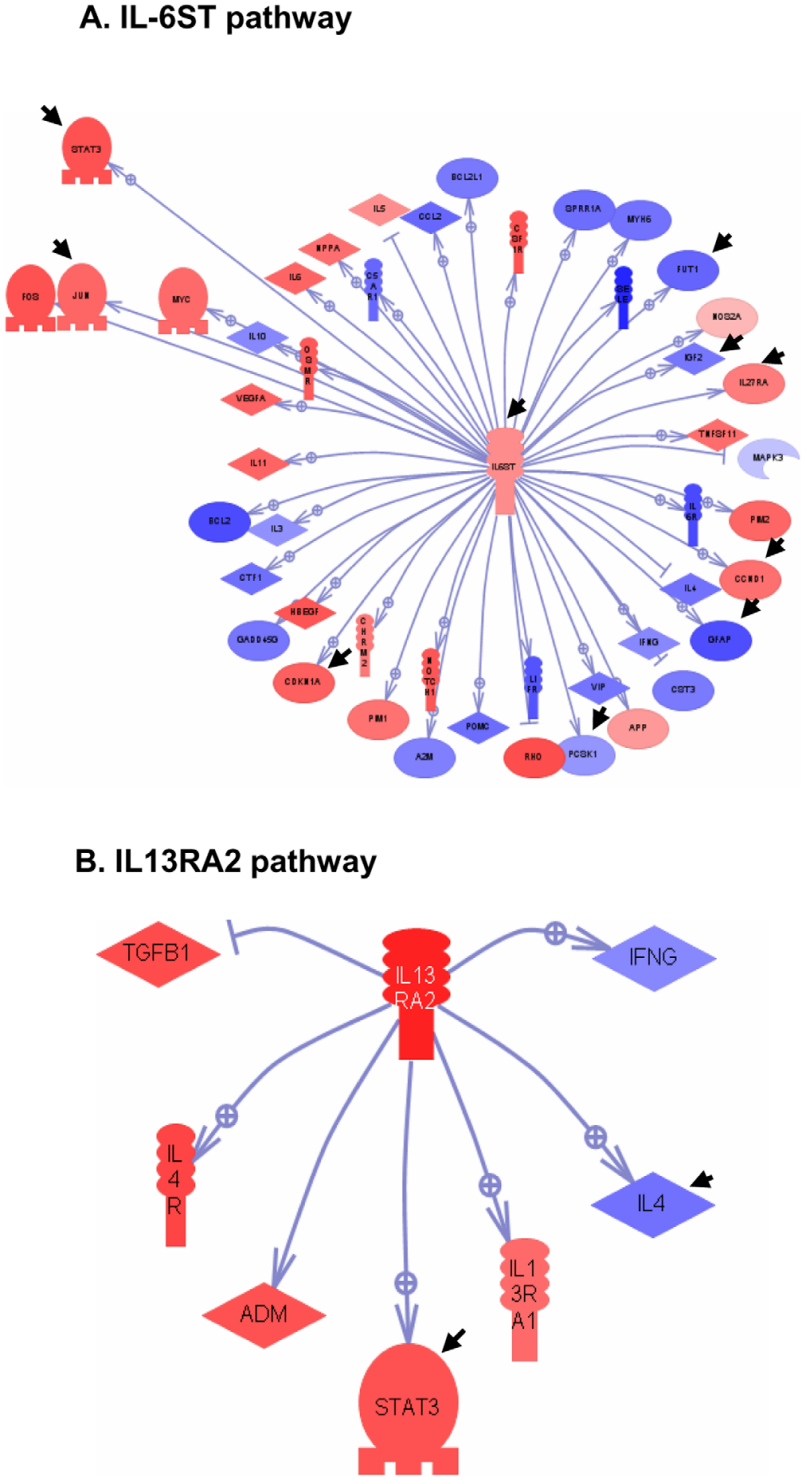
IL-6ST and IL-13RA2 pathways in *C. burnetii*-stimulated BeWo cells. The IL-6ST (A) and IL-13RA2 (B) pathways induced in BeWo cells by *C. burnetii* stimulation were identified using Pathway Studio© software. Up-regulated genes appeared in red and down-regulated genes in blue. Arrowheads represent the differences with TNF stimulation.

The genes found to be modulated in response to *C. burnetii* infection were analyzed with molecular networks using pathway classification and web-based entry tools. This analysis revealed two enriched networks in the response of BeWo trophoblasts to *C. burnetii* infection. The first network consisted of 48 genes and was organized around IL6ST (IL6 signal transducer), also called gp130 (Fig. 5A). In this network, 23 genes were up-regulated in response to *C. burnetii* infection, including IL6ST, STAT3 (signal transducer and activator of transcription, also known as acute phase response factor), JUN (JUN oncogene), IL27RA (interleukin 27 receptor alpha), CCND1 (cyclin D1, also known as parathyroid adenomatosis 1 (PRAD1)) and RHO genes (rhodopsin). Among the 25 genes down-modulated in response to *C. burnetii* infection, some were specific for *C. burnetii*, including IGF2 (insulin-like growth factor 2 or somatomedin A), GFAP (glial fibrially acidic protein) and PCSK1 (proprotein cinvertase subtilisin, also known as kexin type 1). The second network that was markedly up-regulated in response to *C. burnetii* infection was organized around IL13RA2 (IL13Rα2) (Fig. 5B). The network consisted of 7 seven genes, of which five were up-regulated (TGFβ1, IL4R, ADM [adrenomedullin], STAT3, IL13RA1 [IL-13 receptor, alpha 1]) and 2 were down-regulated, including IFN-γ. Taken together, the transcriptional program stimulated by *C. burnetii* was organized in specific networks that were comparable to those induced by TNF stimulation (Fig. S1).

### Transcriptional and protein patterns induced by TNF

Because *C. burnetii* infection stimulated a transcriptional program in BeWo cells in which inflammatory GO terms were enriched, we next investigated whether or not this program was similar to that induced by a typical inflammatory cytokine (TNF) known to activate trophoblast cells [23]. The transcriptional signature induced by TNF was quantitatively and qualitatively different from that induced by *C. burnetii* infection. First, TNF only induced the modulation of 166 genes (142 up-regulated and 24 down-modulated genes), less than half of the total modulated by *C. burnetii* infection (279 and 61 up- and down-regulated, respectively). Second, the majority of genes modulated by TNF were also modulated by *C. burnetii* infection (Fig. 3A), suggesting that *C. burnetii* infection induced an inflammatory response of BeWo cells. In contrast, a very large proportion of the *C. burnetii* infection-modulated genes were *C. burnetii*-specific (Fig. 3A), demonstrating that the response of BeWo cells to *C. burnetii* infection included other major functions.

GO term classification showed that the genes found in clusters 5 and 6 were commonly enriched in response to TNF stimulation and *C. burnetii* infection. They included the genes encoding CXCR6, IFNGR2, MMP12, TNFAIP3, TNFSF10, CD83, FOS (FBJ murine osteosarcoma viral oncogene homolog), S100A9 and CASP5. Again, the increased expression of these genes was confirmed using real time RT-PCR (Fig. 4, C and D). Note that the INDO gene that was down-modulated in response to *C. burnetii* was also depressed in response to TNF stimulation (Fig. 4D). GO term classification also showed major differences in the transcriptional responses induced by *C. burnetii* infection and TNF treatment. Indeed, in cluster 2, the expression of the genes encoding EGR-1, IL4R, MMP9, NDRG1 and TGFβ1 was weakly modulated by TNF treatment when compared to the induction by *C. burnetii* infection (Fig. 4A). In addition, western blotting used to determine EGR-1 protein expression demonstrated that TNF stimulation was unable to up-regulate its expression (Fig. 4B). Taken together, our results showed that TNF induced transcriptional responses in BeWo trophoblasts that were not superimposable with those induced by *C. burnetii* infection.

## Discussion

The aim of this study was to investigate the ability of *C. burnetii* to use trophoblastic cells as a replicative niche. We found that *C. burnetii* is able to infect and replicate within BeWo trophoblasts. The intracellular fate of *C. burnetii* in BeWo trophoblasts is dramatically different from that observed in human monocytes [21] and macrophages [24], in which *C. burnetii* survives but does not replicate. Even in the presence of potent immunoregulatory cytokines, such as IL-10, macrophages are less permissive for *C. burnetii* replication [24] than BeWo trophoblasts. The pertinence of BeWo trophoblasts as a model to study the interplay between trophoblasts and bacterial pathogens is supported by the fact that BeWo cells share several properties with human villous trophoblasts, including morphology, biochemical markers and hormone secretion [25], [26]. It is likely that trophoblasts are a replicative niche for *C. burnetii*, and the results presented here may explain why these bacteria are found in abundance in infected placentas [5], [6].

The intracellular life cycle of *C. burnetii* in BeWo cells was found to be based on replication in phagolysosomes. These results were markedly different from those found in macrophages, where *C. burnetii* resides and replicates within an acidic phagosome that expresses Lamp-1 but not cathepsin D [20]. These findings may be related to those found using non-microbicidal cells, in which *C. burnetii* was shown to reside in phagolysosomes or autophagosomes [27]. It is likely that the intracellular localization of *C. burnetii* is dependent on the cell type used. *Brucella,* which replicates in JEG trophoblasts, is localized within endoplasmic reticulum-derived compartments in epithelial cells [28]; however, it is unable to fuse with lysosomes in macrophages in an acidic compartment [29]. *Mycobacterium tuberculosis* localizes within early phagosomes in macrophages, [30] but in monocyte-derived dendritic cells, it escapes from phagosomes and replicates within the cytosol [31]. It has recently been suggested that the mode of bacterial entry changes the nature of the compartment where *M. tuberculosis* resides without affecting its replication [32]. We cannot rule out the possibility that *C. burnetii* uses different receptors to invade trophoblasts and myeloid cells [21]. Also, we can speculate that the molecular composition of phagolysosomes from macrophages is different from that of non-microbicidal cells. Indeed, the NADPH oxidase, that is involved in the generation of reactive oxygen intermediates, is known to be highly active in stimulated phagocytic cells but is poorly active in non-microbicidal cells [33]. In addition, the inflammatory program induced in myeloid cells is dramatically higher than in non-myeloid cells. The conjunction of the lytic enzymes present in phagolysosomes with the generation of oxidative burst and the production of inflammatory mediators may inhibit *C. burnetii* replication in macrophages while the inability of trophoblast cells to mount an efficient oxidative response may favor *C. burnetii* replication.

*C. burnetii* induced a transcriptional program in BeWo trophoblasts that is organized in 6 clusters of genes based on GO terms. Some clusters shared by both *C. burnetii* and TNF were enriched for inflammation and immunity GO terms. They included several up-regulated genes including TNF-related genes (TNF, TNFAIP3, TNFSF10), IFN-γ-related genes (IFNγR2) and inflammatory mediators (MMP12, S100A9). This inflammatory response is strengthened by the down-modulation of INDO, which is known to exert an anti-inflammatory response that protects developing fetuses from the maternal immune response [34]. The decreased expression of INDO has been associated with cases of miscarriage [35]. These changes in inflammation and immunity GO terms suggest a type 1 cytokine response in trophoblasts. This is consistent with the results we previously obtained with monocytes in which *C. burnetii* stimulated an M1-type program [36]. This is also consistent with the response of different trophoblastic cell lines to the bacterial pathogen *Chlamydia trachomatis* [37]. Other genes organized in clusters were specifically modulated in response to *C. burnetii* but not to TNF. They included genes involved in pregnancy development; hence, *C. burnetii* modulated several genes related to apoptosis. The relationship between apoptosis and pregnancy is complex: a certain level of apoptosis is necessary for placental implantation and protects from obstetrical complications. By contrast, any further increases in the level of apoptosis may compromise normal development of pregnancy [38]. This is distinct from macrophages in which *C. burnetii* inhibits apoptosis [27]. In addition, the apoptosis of *C. burnetii*-infected macrophages by IFN-γ is associated with *C. burnetii* killing [39]. Our results suggest that the induction of apoptosis pathways favors *C. burnetii* replication in BeWo trophoblasts. They also suggest that *C. burnetii* does not interfere with the pro-implantation function of trophoblasts. This latter hypothesis is strengthened by the fact that the transporter SCL16A3, necessary for pre-implantation [40] was up-regulated in response to *C. burnetii* stimulation, whereas TNF stimulation down-modulated its expression. Two genes specifically modulated by *C. burnetii*, EGR-1 and NDRG1, likely play a major role in pregnancy. The expression level of EGR-1 is known to increase in the first trimester of pregnancy and decreases thereafter; thus, it may play a role in trophoblast transcriptional activity and growth [41]. *C. burnetii* stimulated the transcriptional expression of EGR-1, whereas TNF had no effect on the transcription and protein expression levels of EGR-1 (Fig. 4B). The ability to activate EGR-1 has been reported with other pathogens, including group B *Streptococcus* and *Chlamydia pneumoniae* [42], [43]. NDRG1 is a cytoplasmic and nuclear protein involved in stress or hormone responses and cell growth and differentiation. Its expression in primary trophoblasts is stimulated by placental injury [44] and is associated with pregnancy complications [45]. Expression of the NDRG1 gene is known to be stimulated by TGF-β1 [46], and we found that both genes were up-regulated in BeWo trophoblasts stimulated with *C. burnetii*. It is tempting to relate apoptosis, which plays a critical role in the decidual regression that occurs in the end of pregnancy, to TGF-β, which is expressed in the endoterium during decidual basilis regression [46]. As TGF-β is involved in the regulation of apoptosis, we suggest that TGF-β plays an important role in the remodeling of the decidua and governs the apoptotic mechanisms observed during decidua regression [47].

We next tested if the genes modulated in response to *C. burnetii* were organized into networks. We found that *C. burnetii* stimulation induced two pathways: IL6ST and IL13RA2. The IL6 network refers to the molecules that IL6ST (gp130) helps regulate, such as IL-6, IL-11, IL-27 and STAT3. Cytotrophoblasts with an invasive phenotype express IL-6 and its receptor; IL-11 is also expressed by trophoblasts and boosts their migration [48]. IL6ST is essential for placental development because its disruption impairs placentation [49]. A critical link in the IL-6 network was the up-regulation of STAT3 in response to *C. burnetii* stimulation. STAT3 activation has been observed in human placental trophoblasts and trophoblast cell lines in response to T cell products including IL-6 [50]. The inactivation of STAT3 in mice prevents implantation [51]. A major difference between the transcriptional responses of BeWo cells to *C. burnetii* (Fig. 5) and TNF (Fig. S1) stimulation was the down-modulation of STAT3 in response to TNF that contrasted sharply with the up-regulation in response to *C. burnetii*. Our findings suggest that *C. burnetii* does not interfere with the implantation process through STAT3 whereas TNF, which down-modulates STAT3, could compromise normal pregnancy. The network organized around IL13RA2 involves a decoy receptor for IL-13 that exists in membrane, cytoplasmic and soluble forms [52]. The soluble form of IL-13 decoy receptor may interfere with IL-13 signaling, and the membrane form of IL13RA2 attenuates IL-13 responses [53], [54]. The down-modulation of STAT3 is found in several networks, including that of IL13RA2. Studies using tumor cells have shown that the expression of IL13RA2 up-regulates that of STAT3, which ultimately compromises the IL-13 signal [55].

In conclusion, we report here for the first time the ability of *C. burnetii* to infect and replicate within trophoblastic cells. The analysis of the transcriptomic program induced by *C. burnetii* revealed the presence of a non-specific inflammatory response and the increased expression of genes associated with placental development. These results suggest that trophoblasts could be a niche for *C. burnetii* because their activation would not be sufficient to compromise a normal pregnancy. It is likely that the cooperation of trophoblasts and placental immune cells (which are responsive to *C. burnetii*) within the placental tissue impairs the development of pregnancy.

## Materials and Methods

### Ethics Statement

The mice used in our study were handled in strict accordance with the rules of Décret N° 87-848 of 10/19/1987, Paris. The experimental protocol have been reviewed and approved by the Institutional Animal Care and Use Committee of the Université de la Méditerranée (experimentation permit number C13-055-9; personal agreements 13-04 and 13-472).

### Bacteria and cells

*C. burnetii* bacteria (RSA493 Nile Mile strain) were phenotyped [56] and cultured [21] as previously described. The dilacerated spleens of BALB/c mice infected with 10^8^ *C. burnetii * organisms for 7 d were added to L929 cells. Infected cells were sonicated and centrifuged at 300×g for 10 min. Supernatants were collected and centrifuged at 10,000×g for 10 min. The concentration of organisms was determined by Gimenez staining, and the bacterial viability was assessed using the LIVE/DEAD BacLight bacterial viability kit (Molecular Probes). The BeWo (number CCL-98) cell line was obtained from ATCC. Cells were cultured in F-12 Ham medium (Invitrogen) containing 10% FCS, 2 mM L-glutamine, 100 U/ml penicillin and 50 µg/ml streptomycin. Confluent monolayers were trypsinized twice a week.

### *C. burnetii* infection

BeWo trophoblasts (2×10^5^/well) were cultured in flat-bottom 24-well plates for 48 h. They were then incubated with different doses of *C. burnetii* for 4 h. After washing to remove free bacteria (time designated as day 0), cells were cultured for 9 d. Infection was quantified using quantitative real time PCR (qPCR) as previously described [57]. In brief, DNA was extracted using a DNA Mini Kit (Qiagen) and stored in 100 µL aliquots at -20°C. qPCR was performed using 5 µL of DNA extract and the LightCycler FastStart DNA SYBR green system (Roche). The primers F *com1* (5′-GCACTATTTTTAGCCG-GAACCTT-3′) and R *com1* (5′-TTGAGGAGAAAA-ACTGGATTGAGA-3′) amplified a 225-bp fragment of the *C. burnetii com1* gene (GeneBank accession no. AF318146). The specificity of the PCR product was confirmed by sequencing. In each qPCR run, a standard curve was generated using serial dilutions ranging from 10^8^ to 10^4^ copies of the intergenic spacer region and calculated by the Light Cycler 5.32 software (LC-Run version 5.32, Roche).

### Characterization of *C. burnetii* compartment

The compartment in which *C. burnetii* replicated was studied using immunofluorescence, as previously described [20]. BeWo trophoblasts were seeded on glass cover slips prior to infection with *C. burnetii*. After fixation in 3% paraformaldehyde, cells were permeabilized with 0.1% Triton X-100 and incubated with mouse antibodies (Abs) specific for Lamp-1 (1∶1000,Abcam), rabbit Abs directed against cathepsin D (1∶1000, a gift from Dr. Kornfeld, St. Louis, MO) and human Abs specific for *C. burnetii* (1∶8000 dilution) obtained from patients with Q fever with their informed consent. After washing, cells were incubated with secondary fluorescent Abs (Invitrogen), washed, mounted with Mowiol, and analyzed by epifluorescence and laser scanning microscopy. Images were acquired using a confocal microscope (Leica TCS SP5) with a 63X/1.32-0.6 oil objective, an electronic magnification of 1.5× and a resolution of 1024×1024 pixels. Optical sections of fluorescent images were collected at 0.15-µm intervals using the Leica Confocal Software and processed using Adobe Photoshop© V7.0.1. At least 60 cells were examined for each experimental condition. Results were expressed as the percentage of bacteria that co-localized with fluorescent markers with the following color code: the colocalization of bacteria (in red) with Lamp-1 (green) resulted in yellow color; the colocalization of bacteria (red) with cathepsin D (blue) resulted in pink color; the colocalization of bacteria (red) with Lamp-1 (green) and cathepsin D (blue) resulted in white color.

### Microarrays and data analysis

BeWo trophoblasts were stimulated with *C. burnetii* (200 bacteria/cell) or 10 ng/mL human recombinant TNF (R&D Systems) for 6 h, and total RNA was extracted using an RNeasy Mini Kit (Qiagen) and DNase treatment as previously described [58]. The quality of the RNA preparation was assessed using the 2100 Bioanalyzer and the RNA 6000 Nano LabChip kit (Agilent Technologies), and their quantity was assessed using a Nanodrop. The 4X44k Human Whole Genome microarrays (Agilent Technologies) representing 44,000 probes were used as recently described [59]. Sample labeling and hybridization were performed according to protocols specified by the manufacturer (One-Color Microarray-Based Gene Expression Analysis). Three samples per experimental condition were included in the analysis. Slides were scanned at a 5-µm resolution with a G2505B DNA microarray scanner (Agilent Technologies). Image analysis and intra-array signal correction were performed using Agilent Feature Extractor Software© A.9.1.3. Further data processing, analysis and visualization were performed using the Resolver software© (version 6.0, Rosetta Inpharmatics). The hybridized genes (about 30,000 probes) were kept for further statistical analysis. Differentially expressed gene sets consisted of genes matching for an absolute FC>1.4 and a *P*-value <0.01 as determined by the 2-tailed Student's *t* test. Results were expressed as log2 FC.

The GO viewer tool was used to calculate P-values for each GO term as recently reported [59]. In brief, the GO terms were classified by unsupervised hierarchic clustering. Data were entered in the ArrayExpress database following the MIAME procedure and can be retrieved using an accession account (username: Reviewer_E-MEXP-2800 and password: 1278523069329). Gene families were determined using the Resolver Rosetta Biosoftware (http://www.rosettabio.com/products/resolver). The genes were studied using a network generated by PathwayStudioTM© software (Ariadne Genomics).

### Real time RT-PCR and western blot

Total RNA was reverse transcribed using the MMLV-RT kit according to the manufacturer's protocol (Invitrogen). Forward and reverse primers were designed with the free web software Primer3 (see Table S3 for primer sequences). Real time RT-PCR was performed using the Applied Biosystems 7900HT Fast Real-Time PCR System according to the manufacturer's recommendations. Three independent experiments were performed in triplicate. The data are expressed relative to unstimulated cells. A value of 0 indicates that the level of gene expression was similar between stimulated and unstimulated cells. A value higher than 0 indicates that the gene was up-regulated in stimulated cells, and a value less than 0 shows that the gene was down-modulated in stimulated cells.

Western blotting was performed as previously described [60]. In brief, BeWo trophoblasts were stimulated with *C. burnetii* (200 bacteria/cell) for 6 h. Roughly 40 µg of protein was loaded onto sodium dodecyl sulphate-10% polyacrylamide gels and transferred to nitrocellulose sheets. Blots were incubated with a 1∶1,000 dilution of monoclonal antibody (mAb) directed against human EGR-1 (Santa Cruz Biotechnology) or mAb anti-human α-tubulin (Cell Signaling). Proteins were revealed using a 1∶2000 dilution of peroxidase-conjugated F(ab')_2_ anti-mouse immunoglobulin G (IgG; Amersham) for 60 min and enhanced chemiluminescence detection assay.

### Statistical analysis

PCR results are expressed as mean values ± SEM and were compared using the non-parametric Mann-Whitney *U* test. Differences were considered significant when P<0.05.

## Supporting Information

Figure S1 TNF-stimulated networks.
The IL-6ST (A) and IL-13RA2 (B) pathways induced in BeWo cells by TNF were identified using Pathway Studio© software. Up-regulated genes appeared in red and down-modulated genes in blue.
(TIF)

Table S1 Up-regulated genes in response to *C. burnetii*.
In tint, the modulated genes that were also analyzed by qRT-PCR. Cb: *Coxiella burnetii*.
(DOC)

Table S2 Down-modulated genes in response to *C. burnetii*.
In tint, the modulated genes that were also analyzed by qRT-PCR. Cb: *Coxiella burnetii*.
(DOC)

Table S3 Nucleotide sequences of oligonucleotide primers.
(DOC)
