# Supplementary figures and images for: Coxiella burnetii, the Agent of Q Fever, Replicates within Trophoblasts and Induces a Unique Transcriptional Response
Source: PLoS One. 2010 Dec 14;5(12):e15315. doi: 10.1371/journal.pone.0015315 (PMC3001886; doi:10.1371/journal.pone.0015315)

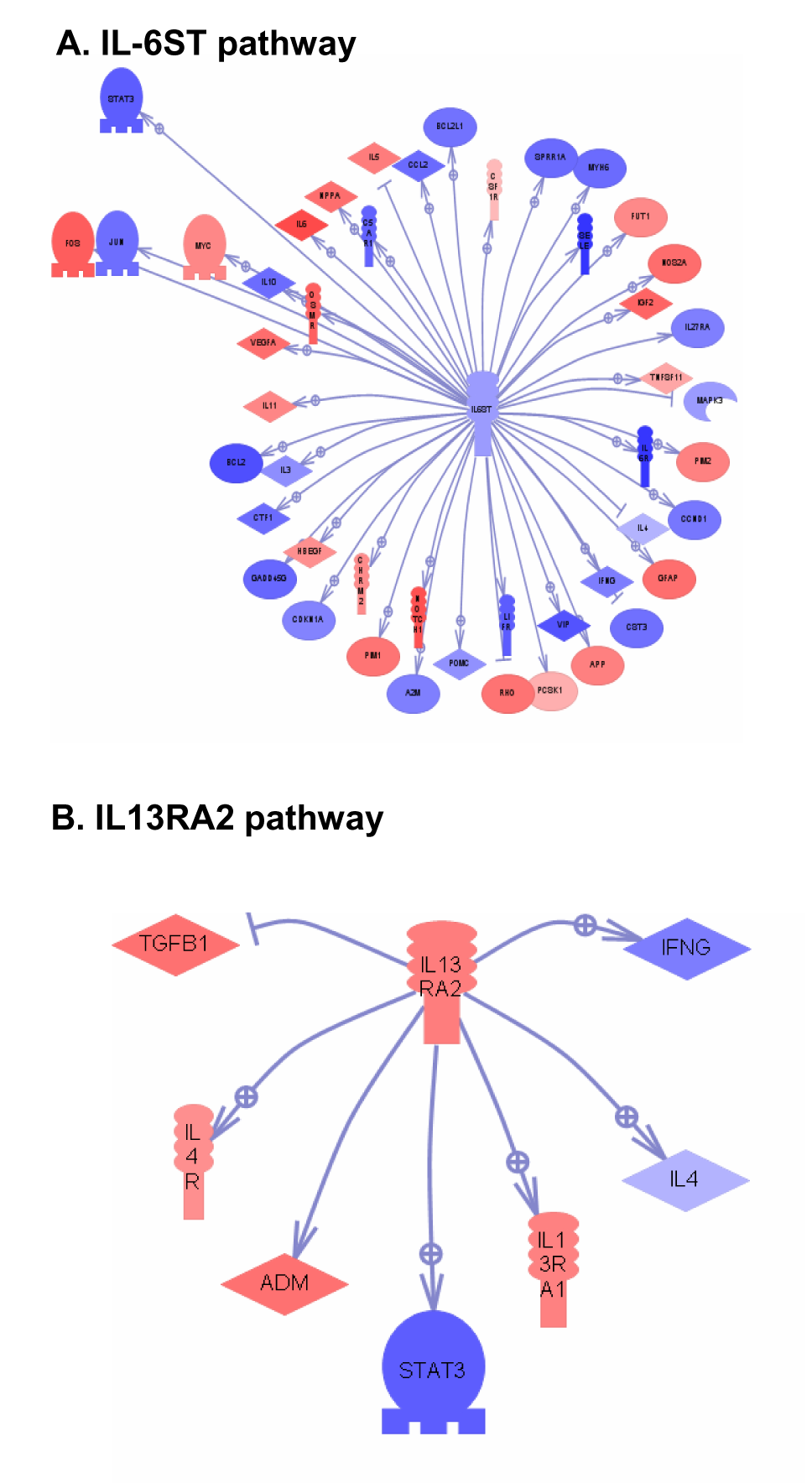

Supplement: Figure S1 — TNF-stimulated networks. The IL-6ST (A) and IL-13RA2 (B) pathways induced in BeWo cells by TNF were identified using Pathway Studio© software. Up-regulated genes appeared in red and down-modulated genes in blue. (TIF) [file pone.0015315.s001.tif]
