# Supplementary material for: Coxiella burnetii, the Agent of Q Fever, Replicates within Trophoblasts and Induces a Unique Transcriptional Response
Source: PLoS One. 2010 Dec 14;5(12):e15315. doi: 10.1371/journal.pone.0015315 (PMC3001886; doi:10.1371/journal.pone.0015315)
Supplement: Table S1 — Up-regulated genes in response to C. burnetii. In tint, the modulated genes that were also analyzed by qRT-PCR. Cb: Coxiella burnetii. (DOC) [file pone.0015315.s002.doc]

| cluster | symbol | gene name | accession # | FC Cb | FC TNF |
| --- | --- | --- | --- | --- | --- |
| 2 | MARCH4 | membrane-associated ring finger (C3HC4) 4 | NM_020814 | 4,96 | -1,25 |
| 2 | TNS4 | tensin 4 | NM_032865 | 4,66 | -1,03 |
| 2 | PTRF | polymerase I and transcript release factor | NM_012232 | 4,65 | 1,02 |
| 2 | SH2D1B | SH2 domain containing 1B | NM_053282 | 4,09 | 1,79 |
| 2 | EGR1 | early growth response 1 | NM_001964 | 3,94 | 1,11 |
| 2 | S100A14 | S100 calcium binding protein A14 | NM_020672 | 3,08 | 1,00 |
| 2 | AQP3 | aquaporin 3 (Gill blood group) | NM_004925 | 3,01 | 1,08 |
| 2 | CHST11 | carbohydrate (chondroitin 4) sulfotransferase 11 | NM_018413 | 2,97 | 1,06 |
| 2 | SPOCD1 | SPOC domain containing 1 | NM_144569 | 2,88 | 1,30 |
| 2 | ITGA2 | integrin, alpha 2 (CD49B, alpha 2 subunit of VLA-2 receptor) | NM_002203 | 2,85 | 1,24 |
| 2 | PLAUR | plasminogen activator, urokinase receptor | NM_001005377 | 2,83 | -1,00 |
| 4 | SERPINE1 | serpin peptidase inhibitor, clade E (nexin, plasminogen activator inhibitor type 1), member 1 | NM_000602 | 2,77 | 2,26 |
| 2 | ESAM | endothelial cell adhesion molecule | NM_138961 | 2,76 | 1,07 |
| 2 | EPHA2 | EPH receptor A2 | NM_004431 | 2,72 | 1,03 |
| 2 | KRT80 | keratin 80 | NM_182507 | 2,66 | -1,03 |
| 2 | CST6 | cystatin E/M | NM_001323 | 2,66 | -1,22 |
| 2 | CYP1A1 | cytochrome P450, family 1, subfamily A, polypeptide 1 | NM_000499 | 2,60 | -1,21 |
| 2 | FLNC | filamin C, gamma (actin binding protein 280) | NM_001458 | 2,56 | 1,10 |
| 2 | DUSP5 | dual specificity phosphatase 5 | NM_004419 | 2,48 | 1,26 |
| 2 | NANOS1 | nanos homolog 1 (Drosophila) | NM_199461 | 2,46 | 1,16 |
| 2 | MYOZ1 | myozenin 1 | NM_021245 | 2,41 | -1,15 |
| 2 | MMP9 | matrix metallopeptidase 9 (gelatinase B, 92kDa gelatinase, 92kDa type IV collagenase) | NM_004994 | 2,38 | 1,08 |
| 2 | DHRS2 | dehydrogenase/reductase (SDR family) member 2 | NM_182908 | 2,29 | -1,08 |
| 2 | LAMB3 | laminin, beta 3 | NM_001017402 | 2,27 | 1,11 |
| 4 | SAT1 | spermidine/spermine N1-acetyltransferase 1 | NM_002970 | 2,24 | 1,39 |
| 2 | ABCC3 | ATP-binding cassette, sub-family C (CFTR/MRP), member 3 | NM_003786 | 2,24 | -1,04 |
| 2 | BIRC7 | baculoviral IAP repeat-containing 7 (livin) | NM_022161 | 2,19 | 1,12 |
| 2 | ITGA3 | integrin, alpha 3 (antigen CD49C, alpha 3 subunit of VLA-3 receptor) | NM_002204 | 2,16 | 1,07 |
| 4 | ADFP | adipose differentiation-related protein | NM_001122 | 2,14 | 1,52 |
| 2 | PDZK1 | PDZ domain containing 1 | NM_002614 | 2,13 | 1,07 |
| 5 | ASB2 | ankyrin repeat and SOCS box-containing 2 | NM_016150 | 2,13 | 2,29 |
| 4 | GPR109B | G protein-coupled receptor 109B | NM_006018 | 2,10 | 1,71 |
| 2 | PALM2-AKAP2 | PALM2-AKAP2 | NM_007203 | 2,07 | 1,18 |
| 4 | SKIL | SKI-like oncogene | NM_005414 | 2,06 | 1,34 |
| 4 | SPRY4 | sprouty homolog 4 (Drosophila) | NM_030964 | 2,05 | 1,37 |
| 2 | TGM2 | transglutaminase 2(C polypeptide, protein-glutamine-γ-glutamyltransferase) | NM_198951 | 2,02 | 1,06 |
| 5 | APCDD1L | adenomatosis polyposis coli down-regulated 1-like | NM_153360 | 2,00 | 3,00 |
| 6 | ALDH3B1 | aldehyde dehydrogenase 3 family, member B1 | NM_000694 | 2,00 | 3,00 |
| 2 | CD52 | CD52 molecule | NM_001803 | 1,98 | 1,07 |
| 4 | BHLHB2 | basic helix-loop-helix domain containing, class B, 2 | NM_003670 | 1,98 | 1,43 |
| 4 | DDIT4 | DNA-damage-inducible transcript 4 | NM_019058 | 1,97 | 1,27 |
| 2 | IL4R | interleukin 4 receptor | NM_000418 | 1,97 | 1,05 |
| 5 | LTB | lymphotoxin beta (TNF superfamily, member 3) | NM_002341 | 1,96 | 1,96 |
| 2 | ELK3 | ELK3, ETS-domain protein (SRF accessory protein 2) | NM_005230 | 1,94 | 1,29 |
| 4 | GPRC5A | G protein-coupled receptor, family C, group 5, member A | NM_003979 | 1,94 | 1,19 |
| 2 | BAMBI | BMP and activin membrane-bound inhibitor homolog (Xenopus laevis) | NM_012342 | 1,92 | 1,01 |
| 4 | MAG1 | lung cancer metastasis-associated protein | NM_032717 | 1,91 | 1,38 |
| 2 | FAM129A | family with sequence similarity 129, member A | NM_052966 | 1,90 | 1,12 |
| 1 | GFOD1 | glucose-fructose oxidoreductase domain containing 1 | NM_018988 | 1,89 | -1,02 |
| 1 | FOSL1 | FOS-like antigen 1 | NM_005438 | 1,89 | -1,02 |
| 2 | GABAR  APL1 | GABA(A) receptor-associated protein like 1 | NM_031412 | 1,88 | 1,19 |
| 1 | APOA2 | apolipoprotein A-II | NM_001643 | 1,87 | -1,1 |
| 5 | MOGAT1 | monoacylglycerol O-acyltransferase 1 | NM_058165 | 1,86 | 2,66 |
| 1 | ANKRD55 | ankyrin repeat domain 55 | NM_024669 | 1,86 | 1,11 |
| 2 | EGR2 | early growth response 2 (Krox-20 homolog, Drosophila) | NM_000399 | 1,85 | 1,10 |
| 1 | EPHA1 | EPH receptor A1 | NM_005232 | 1,83 | 1,03 |
| 2 | C10orf30 | chromosome 10 open reading frame 30 | NM_152751 | 1,80 | 1,14 |
| 4 | KLF10 | Kruppel-like factor 10 | NM_005655 | 1,80 | 1,49 |
| 4 | EDN2 | endothelin 2 | NM_001956 | 1,79 | 1,28 |
| 2 | TNFR  SF12A | tumor necrosis factor receptor superfamily, member 12A | NM_016639 | 1,79 | -1,03 |
| 4 | LIPG | lipase, endothelial | NM_006033 | 1,78 | 1,38 |
| 1 | C4orf26 | chromosome 4 open reading frame 26 | NM_178497 | 1,78 | 1,03 |
| 5 | STS-1 | Cbl-interacting protein Sts-1 | NM_032873 | 1,77 | 1,34 |
| 2 | FRMD6 | FERM domain containing 6 | NM_152330 | 1,77 | 1,15 |
| 1 | CLTB | clathrin, light chain (Lcb) | NM_007097 | 1,77 | -1,16 |
| 5 | LARP6 | La ribonucleoprotein domain family, member 6 | NM_018357 | 1,76 | 1,80 |
| 2 | IRF8 | interferon regulatory factor 8 | NM_002163 | 1,76 | 1,17 |
| 4 | CENTD1 | centaurin, delta 1 | NM_015230 | 1,76 | 1,23 |
| 4 | DUSP1 | dual specificity phosphatase 1 | NM_004417 | 1,74 | 1,22 |
| 1 | PDGFRB | platelet-derived growth factor receptor, beta polypeptide | NM_002609 | 1,74 | 1,03 |
| 1 | CRLF1 | cytokine receptor-like factor 1 | NM_004750 | 1,74 | -1,06 |
| 2 | TMC7 | transmembrane channel-like 7 | NM_024847 | 1,74 | 1,05 |
| 1 | KRT7 | keratin 7 | NM_005556 | 1,73 | -1,19 |
| 5 | ELL2 | elongation factor, RNA polymerase II, 2 | NM_012081 | 1,73 | 1,31 |
| 5 | SLC41A2 | solute carrier family 41, member 2 | NM_032148 | 1,73 | 1,48 |
| 5 | TMEM87B | transmembrane protein 87B | NM_032824 | 1,72 | 1,44 |
| 1 | PHLDA1 | pleckstrin homology-like domain, family A, member 1 | NM_007350 | 1,72 | -1,07 |
| 4 | AK3L2 | adenylate kinase 3-like 2 | NM_001002921 | 1,72 | 1,38 |
| 2 | TGFB1 | transforming growth factor, beta 1 | NM_000660 | 1,72 | 1,19 |
| 4 | SHANK2 | SH3 and multiple ankyrin repeat domains 2 | NM_012309 | 1,71 | 1,22 |
| 2 | GNG11 | guanine nucleotide binding protein (G protein), gamma 11 | NM_004126 | 1,71 | 1,15 |
| 1 | LMCD1 | LIM and cysteine-rich domains 1 | NM_014583 | 1,71 | -1,08 |
| 4 | MAP3K5 | mitogen-activated protein kinase kinase kinase 5 | NM_005923 | 1,70 | 1,33 |
| 5 | IER3 | immediate early response 3 | NM_003897 | 1,70 | 1,54 |
| 2 | RAB7B | RAB7B, member RAS oncogene family | NM_177403 | 1,70 | -1,01 |
| 1 | C2orf59 | chromosome 2 open reading frame 59 | NM_052871 | 1,69 | -1,03 |
| 2 | ETV5 | ets variant gene 5 (ets-related molecule) | NM_004454 | 1,69 | 1,15 |
| 1 | SPHK1 | sphingosine kinase 1 | NM_021972 | 1,69 | -1,02 |
| 2 | NDRG1 | N-myc downstream regulated gene 1 | NM_006096 | 1,68 | 1,14 |
| 1 | MICAL2 | microtubule associated monoxygenase, calponin and LIM domain containing 2 | NM_014632 | 1,68 | -1,04 |
| 5 | TNF | tumor necrosis factor (TNF superfamily, member 2) | NM_000594 | 1,68 | 3,34 |
| 2 | VIM | vimentin | NM_003380 | 1,68 | 1,09 |
| 1 | KRT86 | keratin 86 | NM_002284 | 1,68 | 1,05 |
| 1 | NUAK1 | NUAK family, SNF1-like kinase, 1 | NM_014840 | 1,67 | -1,05 |
| 5 | TACSTD2 | tumor-associated calcium signal transducer 2 | NM_002353 | 1,67 | 1,34 |
| 1 | CASP4 | caspase 4, apoptosis-related cysteine peptidase | NM_033306 | 1,67 | 1,08 |
| 1 | ANKRD57 | ankyrin repeat domain 57 | NM_023016 | 1,67 | 1,07 |
| 1 | FOXO1 | forkhead box O1 | NM_002015 | 1,67 | -1,19 |
| 1 | RAB30 | RAB30, member RAS oncogene family | NM_014488 | 1,66 | 1,16 |
| 1 | KLK6 | kallikrein-related peptidase 6 | NM_001012964 | 1,66 | -1,05 |
| 4 | GPR87 | G protein-coupled receptor 87 | NM_023915 | 1,65 | 1,17 |
| 4 | MAFF | v-maf musculoaponeurotic fibrosarcoma oncogene homolog F (avian) | NM_012323 | 1,64 | 1,16 |
| 5 | SPRED2 | sprouty-related, EVH1 domain containing 2 | NM_181784 | 1,64 | 1,42 |
| 1 | WNT11 | wingless-type MMTV integration site family, member 11 | NM_004626 | 1,64 | 1,04 |
| 1 | TM4SF5 | transmembrane 4 L six family member 5 | NM_003963 | 1,64 | -1,03 |
| 4 | PLEKHA5 | pleckstrin homology domain containing, family A member 5 | NM_019012 | 1,64 | 1,24 |
| 5 | IFNGR2 | interferon gamma receptor 2 (interferon gamma transducer 1) | NM_005534 | 1,64 | 1,46 |
| 1 | MICALCL | MICAL C-terminal like | NM_032867 | 1,64 | 1,00 |
| 5 | ZNF286A | zinc finger protein 286A | NM_020652 | 1,64 | 1,65 |
| 1 | IL1R2 | interleukin 1 receptor, type II | NM_004633 | 1,63 | 1,06 |
| 5 | S100A9 | S100 calcium binding protein A9 | NM_002965 | 1,63 | 1,52 |
| 1 | MMP15 | matrix metallopeptidase 15 (membrane-inserted) | NM_002428 | 1,62 | 1,02 |
| 4 | FAM84B | family with sequence similarity 84, member B | NM_174911 | 1,62 | 1,21 |
| 5 | TGIF1 | TGFB-induced factor homeobox 1 | NM_170695 | 1,62 | 1,19 |
| 2 | F2RL1 | coagulation factor II (thrombin) receptor-like 1 | NM_005242 | 1,62 | 1,10 |
| 4 | SERPINB8 | serpin peptidase inhibitor, clade B (ovalbumin), member 8 | NM_198833 | 1,61 | 1,19 |
| 4 | ZFP36 | zinc finger protein 36, C3H type, homolog (mouse) | NM_003407 | 1,61 | 1,18 |
| 1 | RGS20 | regulator of G-protein signaling 20 | NM_170587 | 1,61 | 1,02 |
| 1 | CSF1R | colony stimulating factor 1 receptor, formerly McDonough feline sarcoma viral (v-fms) oncogene homolog | NM_005211 | 1,6 | 1 |
| 5 | FOS | v-fos FBJ murine osteosarcoma viral oncogene homolog | NM_005252 | 1,60 | 1,39 |
| 5 | STYK1 | serine/threonine/tyrosine kinase 1 | NM_018423 | 1,60 | 1,28 |
| 2 | HSD3B1 | hydroxy-delta-5-steroid dehydrogenase, 3 beta- and steroid delta-isomerase 1 | NM_000862 | 1,60 | 1,13 |
| 4 | ADM | adrenomedullin | NM_001124 | 1,59 | 1,19 |
| 5 | NEDD9 | neural precursor cell expressed, developmentally down-regulated 9 | NM_006403 | 1,59 | 1,48 |
| 5 | KCNA3 | potassium voltage-gated channel, shaker-related subfamily, member 3 | NM_002232 | 1,59 | 1,39 |
| 5 | CPOX | coproporphyrinogen oxidase | NM_000097 | 1,58 | 1,27 |
| 1 | LILRB3 | leukocyte immunoglobulin-like receptor, subfamily B (with TM and ITIM domains), member 3 | NM_006864 | 1,58 | -1,01 |
| 1 | PODXL | podocalyxin-like | NM_005397 | 1,57 | -1,08 |
| 6 | ACOT11 | acyl-CoA thioesterase 11 | NM_147161 | 1,57 | 1,12 |
| 5 | GBP3 | guanylate binding protein 3 | NM_018284 | 1,56 | 1,30 |
| 5 | SLC30A1 | solute carrier family 30 (zinc transporter), member 1 | NM_021194 | 1,56 | 1,50 |
| 3 | SH3RF1 | SH3 domain containing ring finger 1 | NM_020870 | 1,56 | 1,02 |
| 1 | SERINC2 | serine incorporator 2 | NM_178865 | 1,56 | -1,04 |
| 5 | DUSP5P | Homo sapiens dual specificity phosphatase 5 pseudogene (DUSP5P) on chromosome 1. | NR_002834 | 1,56 | 1,21 |
| 1 | LY6K | lymphocyte antigen 6 complex, locus K | NM_017527 | 1,55 | -1 |
| 3 | KIAA1609 | KIAA1609 | NM_020947 | 1,55 | 1,05 |
| 3 | HBEGF | heparin-binding EGF-like growth factor | NM_001945 | 1,54 | 1,05 |
| 6 | PRKCE | protein kinase C, epsilon | NM_005400 | 1,54 | 1,18 |
| 5 | SOHLH1 | spermatogenesis and oogenesis specific basic helix-loop-helix 1 | NM_001012415 | 1,54 | 2,88 |
| 1 | SLC1A7 | solute carrier family 1 (glutamate transporter), member 7 | NM_006671 | 1,54 | -1,02 |
| 1 | EPHB3 | EPH receptor B3 | NM_004443 | 1,54 | -1,17 |
| 5 | CD83 | CD83 molecule | NM_004233 | 1,54 | 1,74 |
| 5 | MAP3K8 | mitogen-activated protein kinase kinase kinase 8 | NM_005204 | 1,54 | 1,56 |
| 5 | AADAT | aminoadipate aminotransferase | NM_016228 | 1,53 | 1,20 |
| 5 | NOTCH1 | Notch homolog 1, translocation-associated (Drosophila) | NM_017617 | 1,53 | 1,61 |
| 6 | RIPK4 | receptor-interacting serine-threonine kinase 4 | NM_020639 | 1,53 | 1,06 |
| 5 | CXCR6 | chemokine (C-X-C motif) receptor 6 | NM_006564 | 1,53 | 1,27 |
| 6 | CASP5 | caspase 5, apoptosis-related cysteine peptidase | NM_004347 | 1,52 | 1,04 |
| 1 | RALB | v-ral simian leukemia viral oncogene homolog B (ras related; GTP binding protein) | NM_002881 | 1,52 | -1,08 |
| 3 | FBLIM1 | filamin binding LIM protein 1 | NM_017556 | 1,52 | -1,00 |
| 5 | GPRC5B | G protein-coupled receptor, family C, group 5, member B | NM_016235 | 1,52 | 2,22 |
| 3 | C4orf18 | chromosome 4 open reading frame 18 | NM_016613 | 1,51 | 1,06 |
| 5 | TNFAIP3 | tumor necrosis factor, alpha-induced protein 3 | NM_006290 | 1,51 | 2,37 |
| 5 | PLEKHC1 | pleckstrin homology domain containing, family C (with FERM domain) member 1 | NM_006832 | 1,51 | 1,15 |
| 5 | TRIM16 | tripartite motif-containing 16 | NM_006470 | 1,51 | 1,17 |
| 5 | CDYL | chromodomain protein, Y-like | NM_170752 | 1,50 | 1,46 |
| 5 | FAM59A | family with sequence similarity 59, member A | NM_022751 | 1,50 | 1,12 |
| 6 | OSBPL10 | oxysterol binding protein-like 10 | NM_017784 | 1,50 | 1,13 |
| 3 | FAM139A | family with sequence similarity 139, member A | NM_173678 | 1,50 | 1,06 |
| 5 | MMP12 | matrix metallopeptidase 12 (macrophage elastase) | NM_002426 | 1,49 | 1,37 |
| 3 | C13orf15 | chromosome 13 open reading frame 15 | NM_014059 | 1,49 | -1,01 |
| 5 | PLCL2 | phospholipase C-like 2 | NM_015184 | 1,49 | 1,18 |
| 6 | ECM1 | extracellular matrix protein 1 | NM_004425 | 1,48 | 1,14 |
| 3 | AJAP1 | adherens junction associated protein 1 | NM_018836 | 1,48 | -1,06 |
| 5 | LRRC32 | leucine rich repeat containing 32 | NM_005512 | 1,48 | 1,88 |
| 6 | ERRFI1 | ERBB receptor feedback inhibitor 1 | NM_018948 | 1,48 | 1,17 |
| 5 | OSMR | oncostatin M receptor | NM_003999 | 1,48 | 1,36 |
| 5 | HES1 | hairy and enhancer of split 1, (Drosophila) | NM_005524 | 1,48 | 1,29 |
| 6 | C19orf21 | chromosome 19 open reading frame 21 | NM_173481 | 1,48 | 1,16 |
| 6 | SLTM | SAFB-like, transcription modulator | NM_024755 | 1,47 | 1,18 |
| 5 | SGK3 | serum/glucocorticoid regulated kinase family, member 3 | NM_013257 | 1,47 | 1,29 |
| 3 | WIPF1 | WAS/WASL interacting protein family, member 1 | NM_003387 | 1,47 | 1,08 |
| 3 | IER5L | immediate early response 5-like | NM_203434 | 1,47 | -1,06 |
| 5 | IL23A | interleukin 23, alpha subunit p19 | NM_016584 | 1,47 | 1,79 |
| 3 | GOLT1A | golgi transport 1 homolog A (S. cerevisiae) | NM_198447 | 1,47 | -1,12 |
| 6 | CARD10 | caspase recruitment domain family, member 10 | NM_014550 | 1,47 | 1,10 |
| 3 | MMP1 | matrix metallopeptidase 1 (interstitial collagenase) | NM_002421 | 1,47 | 1,03 |
| 5 | HIVEP2 | human immunodeficiency virus type I enhancer binding protein 2 | NM_006734 | 1,46 | 1,71 |
| 5 | PPP1R3B | protein phosphatase 1, regulatory (inhibitor) subunit 3B | NM_024607 | 1,46 | 1,25 |
| 5 | TRHDE | thyrotropin-releasing hormone degrading enzyme | NM_013381 | 1,46 | 1,62 |
| 6 | KLF7 | Kruppel-like factor 7 (ubiquitous) | NM_003709 | 1,46 | 1,07 |
| 5 | TNFSF10 | tumor necrosis factor (ligand) superfamily, member 10 | NM_003810 | 1,45 | 1,31 |
| 3 | CHST7 | carbohydrate (N-acetylglucosamine 6-O) sulfotransferase 7 | NM_019886 | 1,45 | -1,02 |
| 6 | SAMD11 | sterile alpha motif domain containing 11 | NM_152486 | 1,45 | 1,18 |
| 6 | HAS3 | hyaluronan synthase 3 | NM_005329 | 1,45 | 1,08 |
| 3 | UPP1 | uridine phosphorylase 1 | NM_181597 | 1,45 | -1,02 |
| 3 | MBOAT2 | membrane bound O-acyltransferase domain containing 2 | NM_138799 | 1,44 | 1,03 |
| 6 | MST150 | MSTP150 | NM_032947 | 1,44 | 1,15 |
| 3 | INF2 | inverted formin, FH2 and WH2 domain containing | NM_032714 | 1,44 | -1,02 |
| 3 | BCL2A1 | BCL2-related protein A1 | NM_004049 | 1,43 | -1,06 |
| 5 | FAM107B | family with sequence similarity 107, member B | NM_031453 | 1,43 | 1,30 |
| 5 | COL5A1 | collagen, type V, alpha 1 | NM_000093 | 1,42 | 1,17 |
| 6 | MANEAL | mannosidase, endo-alpha-like | NM_152496 | 1,42 | 1,02 |
| 5 | INPP5A | inositol polyphosphate-5-phosphatase, 40kDa | NM_005539 | 1,42 | 1,24 |
| 1 | SLC16A3 | solute carrier family 16, member 3 (monocarboxylic acid transporter 4) | NM_004207 | 1,42 | -1,01 |
| 5 | C11orf61 | chromosome 11 open reading frame 61 | NM_024631 | 1,42 | 1,29 |
| 6 | ARHGAP26 | Rho GTPase activating protein 26 | NM_015071 | 1,41 | 1,22 |
| 3 | HVCN1 | hydrogen voltage-gated channel 1 | NM_032369 | 1,41 | 1,12 |
| 6 | TMCC1 | transmembrane and coiled-coil domain family 1 | NM_001017395 | 1,41 | 1,18 |
| 5 | EPB41L4B | erythrocyte membrane protein band 4.1 like 4B | NM_018424 | 1,41 | 1,30 |
| 6 | LAD1 | ladinin 1 | NM_005558 | 1,40 | 1,03 |
| 1 | CTNNAL1 | catenin (cadherin-associated protein), alpha-like 1 | NM_003798 | 1,4 | 1,09 |
