# Supplementary material for: Coxiella burnetii, the Agent of Q Fever, Replicates within Trophoblasts and Induces a Unique Transcriptional Response
Source: PLoS One. 2010 Dec 14;5(12):e15315. doi: 10.1371/journal.pone.0015315 (PMC3001886; doi:10.1371/journal.pone.0015315)
Supplement: Table S2 — Down-modulated genes in response to C. burnetii. In tint, the modulated genes that were also analyzed by qRT-PCR. Cb: Coxiella burnetii. (DOC) [file pone.0015315.s003.doc]

| Cluster Nb | Symbol | Gene Name | Accession # | FC Cb | FC TNF |
| --- | --- | --- | --- | --- | --- |
| 6 | RUN  DC2B | RUN domain containing 2B | NM_001012391 | -1,40 | -1,29 |
| 6 | GH1 | growth hormone 1 | NM_000515 | -1,40 | -1,24 |
| 6 | ARHGA  P22 | Rho GTPase activating protein 22 | NM_021226 | -1,41 | -1,25 |
| 6 | NKG7 | natural killer cell group 7 sequence | NM_005601 | -1,41 | -1,45 |
| 6 | MYO1C | myosin IC | NM_033375 | -1,41 | 1,07 |
| 6 | TRERF1 | transcriptional regulating factor 1 | NM_033502 | -1,41 | -1,18 |
| 6 | NAV2 | neuron navigator 2 | NM_182964 | -1,41 | 1,20 |
| 6 | ETV7 | ets variant gene 7 (TEL2 oncogene) | NM_016135 | -1,41 | -1,17 |
| 6 | PIP5K1B | phosphatidylinositol-4-phosphate 5-kinase, type I, beta | NM_003558 | -1,42 | 1,15 |
| 6 | CSH1 | chorionic somatomammotropin hormone 1 (placental lactogen) | NM_001317 | -1,42 | -1,24 |
| 6 | CPT1A | carnitine palmitoyltransferase 1A | NM_001876 | -1,43 | -1,14 |
| 6 | MUC15 | mucin 15, cell surface associated | NM_145650 | -1,43 | 1,16 |
| 6 | CSHL1 | chorionic somatomammotropin hormone-like 1 | NM_022579 | -1,45 | -1,17 |
| 6 | CREB3L4 | cAMP responsive element binding protein 3-like 4 | NM_130898 | -1,45 | -1,05 |
| 6 | RPUSD3 | RNA pseudouridylate synthase domain containing 3 | NM_173659 | -1,47 | -1,44 |
| 6 | MXD4 | MAX dimerization protein 4 | NM_006454 | -1,47 | -1,27 |
| 6 | TACC1 | transforming, acidic coiled-coil containing protein 1 | NM_006283 | -1,47 | -1,02 |
| 6 | IL21R | interleukin 21 receptor | NM_181078 | -1,47 | -1,18 |
| 6 | INDO | indoleamine-pyrrole 2,3 dioxygenase | NM_002164 | -1,48 | -1,49 |
| 6 | ACVR2B | Homo sapiens activin A receptor, type IIB (ACVR2B), mRNA. | NM_001106 | -1,48 | -1,45 |
| 6 | MXD3 | MAX dimerization protein 3 | NM_031300 | -1,49 | -1,24 |
| 5 | KCNJ6 | potassium inwardly-rectifying channel, subfamily J, member 6 | NM_002240 | -1,49 | 1,45 |
| 6 | ACTA1 | Homo sapiens actin, alpha 1, skeletal muscle (ACTA1), mRNA. | NM_001100 | -1,51 | -1,07 |
| 6 | LDHAL6A | lactate dehydrogenase A-like 6A | NM_144972 | -1,53 | -1,82 |
| 6 | CPZ | carboxypeptidase Z | NM_001014448 | -1,55 | 1,03 |
| 6 | FAM46B | family with sequence similarity 46, member B | NM_052943 | -1,55 | -1,31 |
| 5 | BTN3A3 | butyrophilin, subfamily 3, member A3 | NM_006994 | -1,56 | 1,52 |
| 6 | SLC7A11 | solute carrier family 7, (cationic amino acid transporter, y+ system) member 11 | NM_014331 | -1,61 | 1,44 |
| 6 | ACTA2 | actin, alpha 2, smooth muscle, aorta | NM_001613 | -1,66 | -1,11 |
| 6 | PLEK2 | pleckstrin 2 | NM_016445 | -1,68 | -1,39 |
| 6 | HIST1H4E | histone cluster 1, H4e | NM_003545 | -1,69 | -1,73 |
| 5 | SORBS2 | Sorbin, SH3 domain containing 2 | NM_021069 | -1,72 | 1,44 |
| 6 | MYCB  PAP | MYCBP associated protein | NM_032133 | -1,72 | -1,33 |
| 6 | CASP12 | Homo sapiens caspase 12 (CASP12)  on chromosome 11. | NR_000035 | -1,80 | -1,70 |
| 6 | LZTS1 | leucine zipper, putative tumor suppressor 1 | NM_021020 | -1,83 | 1,06 |
