# Supplementary material for: Coxiella burnetii, the Agent of Q Fever, Replicates within Trophoblasts and Induces a Unique Transcriptional Response
Source: PLoS One. 2010 Dec 14;5(12):e15315. doi: 10.1371/journal.pone.0015315 (PMC3001886; doi:10.1371/journal.pone.0015315)
Supplement: Table S3 — Nucleotide sequences of oligonucleotide primers. (DOC) [file pone.0015315.s004.doc]

| **Symbol** | **Accession number** | **Primers sequences** |
| --- | --- | --- |
| ERG1 | NM_001964 | LEFT PRIMER taaaggacaggaggaggagatg RIGHT PRIMER tagcattgaagggagcaagg |
| IL4R | NM_000418 | LEFT PRIMER gctatgactaaagcagggacaa RIGHT PRIMER cactctcctcagcaaacacaga |
| MMP9 | NM_004994 | LEFT PRIMER caccaccacaacatcacctatt RIGHT PRIMER cagggaccacaactcgtca |
| AQP3 | NM_004925 | LEFT PRIMER caacgaggaagagaatgtgaag RIGHT PRIMER cgtggggtgagggtagatag |
| NDRG1 | NM_006096 | LEFT PRIMER cttttcctgctccctaaccttt RIGHT PRIMER ggactacttcctcctcccaact |
| TGFB1 | NM_000660 | LEFT PRIMER ggtcgggagaagaggaaaaa RIGHT PRIMER cgaggtctggggaaaagtc |
| CXCR6 | NM_006564 | LEFT PRIMER atctcaggttctccttgattgg RIGHT PRIMER cacttgttttcactgctgctct |
| IFNGR2 | NM_005534 | LEFT PRIMER aggcacaactgctttggaac RIGHT PRIMER atctgtaatgggatgcttggtg |
| MMP12 | NM_002426 | LEFT PRIMER acatcaacacatttcgcctctc RIGHT PRIMER cggtagtgacagcatcaaaact |
| TNFAIP3 | NM_006290 | LEFT PRIMER ttggagatgagatagggaagga RIGHT PRIMER tgtaagccagcaacagaaagag |
| TNFSF10 | NM_003810 | LEFT PRIMER ggctaactgacctggaaagaaa RIGHT PRIMER tttggttgtggctgctctact |
| CD 83 | [NM_004233](http://www.ncbi.nlm.nih.gov/entrez/query.fcgi?db=Nucleotide&cmd=Search&term=NM_004233&doptcmdl=GenBank) | LEFT PRIMER : ctcctgggtcaagttattggag  RIGHT PRIMER:ggtagtgtttcggatcttcagg |
| FOS | [NM_0052](http://www.ncbi.nlm.nih.gov/entrez/query.fcgi?db=Nucleotide&cmd=Search&term=NM_004233&doptcmdl=GenBank)52 | LEFT PRIMER: tgttcctggcaatagtgtgttc  RIGHT PRIMER: gaacattcagaccacctcaaca |
| S100A9 | [NM_002965](http://www.ncbi.nlm.nih.gov/entrez/query.fcgi?db=Nucleotide&cmd=Search&term=NM_004233&doptcmdl=GenBank) | LEFT PRIMER: ctaagaccacagtggccaagat  RIGHT PRIMER: cacagccaagacagtttgacat |

Table S3
